# Correlation between ischemia-modified albumin level and coronary collateral circulation

**DOI:** 10.1186/s12872-020-01543-9

**Published:** 2020-07-08

**Authors:** Xin Chen, Yan Lin, Lihua Tian, Zhiquan Wang

**Affiliations:** Department of Cardiology, Zhongnan Hospital of Wuhan University, Wuhan, 430071 Hubei China

**Keywords:** Coronary artery disease;chronic total occlusion;collateral circulation;ischemia modified albumin

## Abstract

**Objective:**

To investigate the correlation between ischemia-modified albumin (IMA) levels and coronary collateral circulation (CCC) in patients with chronic total occlusive (CTO).

**Methods:**

Coronary angiography was performed in the Department of Cardiology, Zhongnan Hospital of Wuhan University from 2017 to 08 to 2019–02 to identify 128 patients with CTO lesions in at least one major coronary artery. According to the Rentrop evaluation criteria, the degree of CCC formation was divided into the poor CCC formation group (Rentrop0–1 grade,*n* = 69) and the good CCC formation group (Rentrop2–3 grade,*n* = 59). The IMA level of the patients was measured using an albumin-cobalt binding assay. The general data, routine blood panel, total bilirubin (TBIL), blood lipids, uric acid (UA), left ventricular ejection fraction (LVEF) and other indicators of the patients were recorded and analyzed while assessing the patients’ blood vessel occlusion.

**Results:**

The proportion of platelet count and diabetes in the poor CCC group was higher than that in the good CCC group (*P* < 0.05). The ratio of ischemia-modified albumin and total bilirubin in the poor CCC group was lower than that in the good CCC group (*P* < 0.05). Multivariate logistic regression analysis showed that ischemia-modified albumin was positively correlated with CCC formation [OR = 1.190,95% CI (1.092–1.297),*P* < 0.001], while diabetes was negatively correlated with CCC formation [OR = 0.285,95% CI (0.094–0.864), *P* < 0.05]. Ischemic modified albumin predicted good formation of CCC according to the ROC curve, and the area under the ROC curve was 0.769(95% CI,0.686–0.851, P<0.001); the optimal cut-off value was 63.35 KU/L, and the sensitivity was 71.2%,specificity is 71%.

**Conclusion:**

The IMA level is closely related to good formation of CCC. Higher IMA levels can be used as an effective predictor of good CCC formation in patients with CTO.

## Introduction

Chronic total occlusion (CTO) is defined as the presence of TIMI 0 flow within an occluded arterial segment for more than 3 months [1]. Coronary collateral circulation (CCC) is a small blood flow channel between different coronary arteries or different segments of the same coronary artery. Usually, these collateral blood flows are extremely small under physiological conditions and are not involved in the blood circulation of the coronary arteries. Under the stimulation of chronic or repeated myocardial ischemia, the collateral vessels gradually open up and result into functional collateral circulation [2]. Well-developed CCC protects the ischemic myocardium, increases coronary blood flow reserve, helps protect left ventricular function, mitigates myocardial infarcts and improves survival [3, 4]. In CTO percutaneous coronary intervention (PCI), the collateral vessels allow visualization of the distal coronary bed beyond the occlusive segment and provide retrograde access to the occluded vessel to facilitate recanalization [5]. Zhu et al. [6] performed a meta-analysis of a study and showed that compared with the poor collateralized group, the well-collateralized group had obviously reduced mortality and a lower incidence of reinfarction and MACEs.

Ischemia-modified albumin (IMA) is characterized by a change in the N-terminal sequence of the albumin amino group in human serum albumin (HSA) during tissue ischemia, resulting in a decrease in the ability to bind to free metal ions such as cobalt, copper and nickel [7]. IMA is currently considered a new biomarker for myocardial ischemia that can be elevated in the early stages of irreversible necrosis of cardiomyocytes. Compared with other myocardial injury biochemicals in patients with acute coronary syndrome, Markers can be detected earlier and with higher sensitivity [8, 9]. IMA levels also increase during myocardial ischemia-reperfusion injury, which is associated with oxidative stress induced by cardiac and extracardiac events.

Current studies have shown that IMA can sensitively reflect early myocardial ischemia, but the relationship between IMA levels and CCC in CTO is uncertain. This study aimed to explore the correlation between IMA and CCC.

## Materials and methods

Coronary angiography was performed in the Department of Cardiology, Zhongnan Hospital of Wuhan University from 2017 to 08 to 2019–02 and identified 128 patients with CTO lesions in at least one major coronary artery (LAD,LCX or RCA) who were evaluated by two experienced interventional cardiologists. **Inclusion criteria**: According to the CTO diagnostic criteria of the American College of Cardiology Foundation (ACCF)/American Heart Association (AHA)/the Society for Cardiovascular Angiography and Interventions (SCAI) CTO in 2013 [10]:CTO was diagnosed on the basis of atherosclerotic lesions when the main coronary artery has at least one stenosis (> 90%) due to thrombosis and repeated organization and the course of the disease is more than 3 months. Coronary heart disease diagnostic criteria were based on the guidelines of the American Heart Association Foundation/American Heart Association for the management of coronary heart disease. Diabetes was diagnosed according to the 2018 Standards of Medical Care in Diabetes recommended by the American Diabetes Association (ADA) [11]: 2 h PG ≥200 mg/dL (11.1 mmol/L) during OGTT;a test performed as described by the WHO, using a glucose load containing the equivalent of 75 g anhydrous glucose dissolved in water; A1C ≥6.5% (48 mmol/mol) baesd on a test performed in a laboratory using a method that is NGSP certified and standardized to the DCCT assay; or in a patient with classic symptoms of hyperglycemia or hyperglycemic crisis, a random plasma glucose ≥200 mg/dL (11.1 mmol/L). According to the 2018 ESC/ESH Guidelines for the management of arterial hypertension [12], arterial hypertension was defined as a measured SBP value of ≥140 mmHg and/or a DBP level ≥ 90 mmHg. **Exclusion criteria:** Previous acute myocardial infarction within 3 months, PCI and/or CABG within 3 months, coronary myocardial bridge and/or malformation, severe heart failure (LVEF< 30%), severe valvular disease, cardiomyopathy, severe liver or kidney dysfunction, severe infection, severe anemia, and malignant tumors. All subjects provided informed consent for the study, which was approved by the Ethics Committee of Zhongnan Hospital, Wuhan University.

### Clinical data

The patients’names, sex, age, history [coronary heart disease (all medical history was over 3 months), hypertension, diabetes mellitus, smoking, alcohol consumption], drug use (aspirin, statins, ACEI/ARB), and data on CTO lesions in blood vessels were recorded LVEF was recorded by echocardiography after admission; venous blood samples were collected on the morning of admission after the participants had fasted for at least 8 h; IMA levels were measured by albumin-cobalt binding assay; and routine blood, blood lipids, total bilirubin and uric acid levels were also assessed.

### CCC evaluation and grouping

Two experienced interventional cardiologists evaluated the results of coronary angiography to determine the formation of CCC in patients who met the criteria for coronary angiography via radial or femoral arteries. Classification of CCC was performed according to the Cohen-Rentrop standard [5]: Grade 0, no visible filling of any collateral channel; Grade 1, filling of the side branches of the occluded artery, with no dye reaching the epicardial segment; Grade 2, partial filling of the epicardial vessel; and Grade 3, complete filling of the epicardial vessel by collaterals. The patients were further divided into the poor CCC group (Rentrop 0–1) and the good CCC group (Rentrop 2–3).

### Statistical analysis

IBM SPSS 22.0 software (IBM Corporation, Armonk, New York, United States) was used for statistical analysis. The measurement data conforming to a normal distribution are expressed as, and the comparisons between the two groups were performed by independent sample t-test. Non-normally distributed measurement data are expressed as M (P25, 75), and comparisons between the two groups were performed by Mann-Whitney U test. The counting data are expressed as frequency (%), and the comparisons between groups were performed by x^2^-test. The comparison of measurement data among Rentrop classification groups was based on one-way ANOVA. A multivariate logistic regression model was used to analyze the factors affecting the formation of CCC. The receiver operating characteristic (ROC) curve was used to describe the predictive value of the research indicators, with *P* < 0.05 indicating statistical significance.

## Results

### Univariate analysis

The proportion of patients with platelet count and diabetes in the poor CCC group was higher than that in the good CCC group (*P* < 0.05);the levels of IMA and total bilirubin in the poor CCC group were lower than those in the good CCC group (P < 0.05). There were no significant differences in other indicators between the two groups (*P* > 0.05) (Table 1).

**Table 1:** Patient baseline characteristics[±s, n(%)]

Variables	Good CCC (n = 59)	Poor CCC (n = 69)	P
Age (years)	60.92 ± 11.61	61.28 ± 11.61	0.861
Male	45 (76.3)	45 (65.2)	0.172
Coronary heart disease	10 (16.9)	5 (7.2)	0.089
Hypertension	39 (66.1)	35 (50.7)	0.079
Diabetes mellitus	9 (15.3)	24 (34.8)	0.012
Smoking	25 (42.4)	27 (39.1)	0.710
Drinking	10 (16.9)	14 (20.3)	0.629
Statins	8 (13.6)	3 (4.3)	0.064
Aspirin	6 (10.2)	3 (4.3)	0.300
ACEI/ARB	10 (16.9)	16 (23.2)	0.382
Platelet(× 10^9/L)	195.97 ± 55.46	218.30 ± 71.62	0.049
Neutrophil(× 10^9/L)	5.45 (3.70,6.21)	6.48 (3.75,8.78)	0.091
Lymphocyte(×10^9/L)	1.64 (1.12,1.89)	1.41 (1.02,1.79)	0.105
Monocyte(×10^9/L)	0.66 (0.37,0.64)	0.56 (0.36,0.63)	0.636
Total cholesterol (mmoL/L)	4.79 (3.72,5.30)	4.43 (3.58,5.21)	0.247
Triglyceride (mmoL/L)	2.14 (1.06,2.19)	1.81 (0.99,2.22)	0.602
HDL-C (mmoL/L)	0.98 (0.82,1.11)	1.88 (0.78,1.10)	0.482
LDL-C (mmoL/L)	2.94 ± 1.06	2.80 ± 0.89	0.418
LP(a)(mg/L)	214.96 (76.30,289.10)	202.16 (56.85,244.45)	0.324
Total bilirubin (μmoL/L)	15.04 ± 5.63	12.58 ± 5.01	0.010
IMA (KU/L)	66.31 ± 5.94	60.51 ± 5.45	< 0.001
Uric acid (μmoL/L)	373.61 ± 111.65	368.76 ± 108.56	0.804
LVEF(%)	60.67 ± 10.28	62.34 ± 8.96	0.327
Occlusive vessels			
Multivessel	13 (22.0)	8 (11.6)	0.112
LAD	25 (42.4)	23 (33.3)	0.292
LCX	4 (6.8)	12 (17.4)	0.070
RCA	17 (28.2)	26 (37.7)	0.290

CCC:coronary collateral circulation;ACEI:angiotensin converting enzyme inhibitors;ARB:Angiotensin II receptor antagonist;HDL-C:high density lipoprotein cholesterol;LDL-C:low density lipoprotein cholesterol;LP(a):lipoprotein a;IMA:ischemic modified albumin;LVEF:left ventricular ejection fraction;Occlusive vessels:more than two main coronary arteries;LAD:left anterior descending artery;LCX:left circumflex;RCA:Right coronary artery

### Multivariate logistic regression analysis

With the formation of CCC as the dependent variable and the factors with *P* < 0.1 in the univariate analysis as the independent variables, multivariate logistic regression analysis showed that diabetes was negatively correlated with CCC [OR = 0.285 95% CI (0.094–0.864) *P* < 0.05] and IMA was positively correlated with CCC [OR = 1.190 95% CI (1.092–1.297) *P* < 0.001]. The results showed that the level of IMA and diabetes were independent factors affecting CCC (Table 2).

**Table 2:** Multivariate logistic regression analysis

Variables	OR	95%CI	P
Coronary heart disease	1.842	0.344–9.874	0.476
Hypertension	1.752	0.703–4.367	0.229
Diabetes mellitus	0.285	0.094–0.864	0.027
Statins	1.605	0.247–10.446	0.620
Platelet	0.995	0.987–1.003	0.242
Neutrophil	0.931	0.800–1.085	0.361
Total cholesterol	1.077	0.989–1.172	0.089
IMA	1.190	1.092–1.297	< 0.001
LCX	0.341	0.085–1.373	0.130

### Rentrop grading correlation analysis

**Fig. 1:**
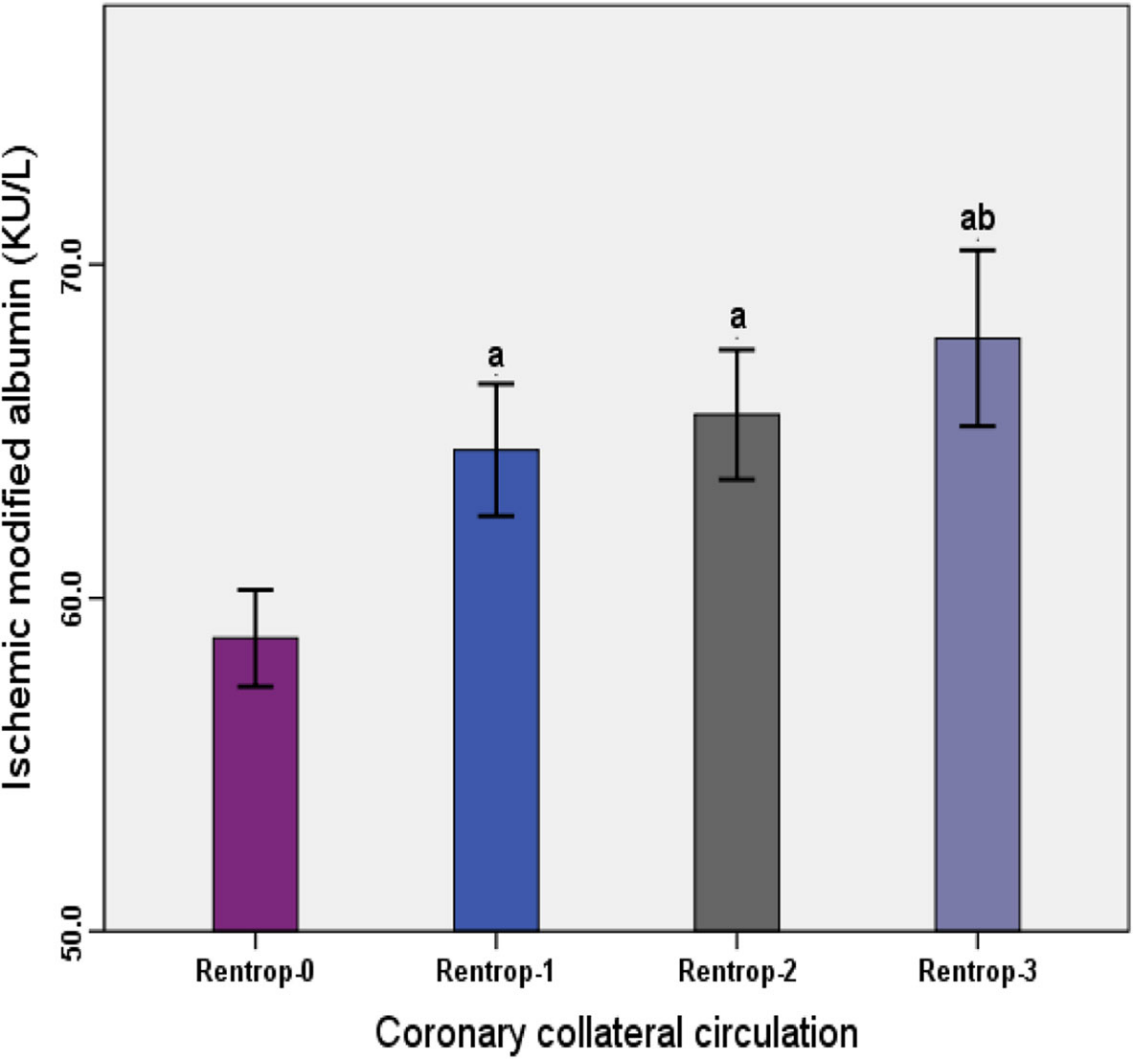
Comparison of IMA in various Rentrop grades (comparison with Rentrop0,^a^*P* < 0.001,comparison with Rentrop1,^b^*P* < 0.05)

The level of IMA at Rentrop 0 (58.79 ± 5.00) was lower than that at Rentrop 1 (64.44 ± 4.36), Rentrop 2 (65.50 ± 5.93) and Rentrop 3 (67.79 ± 5.79) (*P* < 0.001). There was no significant difference in the level of IMA between Rentrop 1 and Rentrop 2 and Rentrop 3 levels (*P* > 0.05). There was no significant difference in the level of IMA between Rentrop 2 and Rentrop 3 (P > 0.05) (Fig. 1).

### ROC curve results

**Fig. 2:**
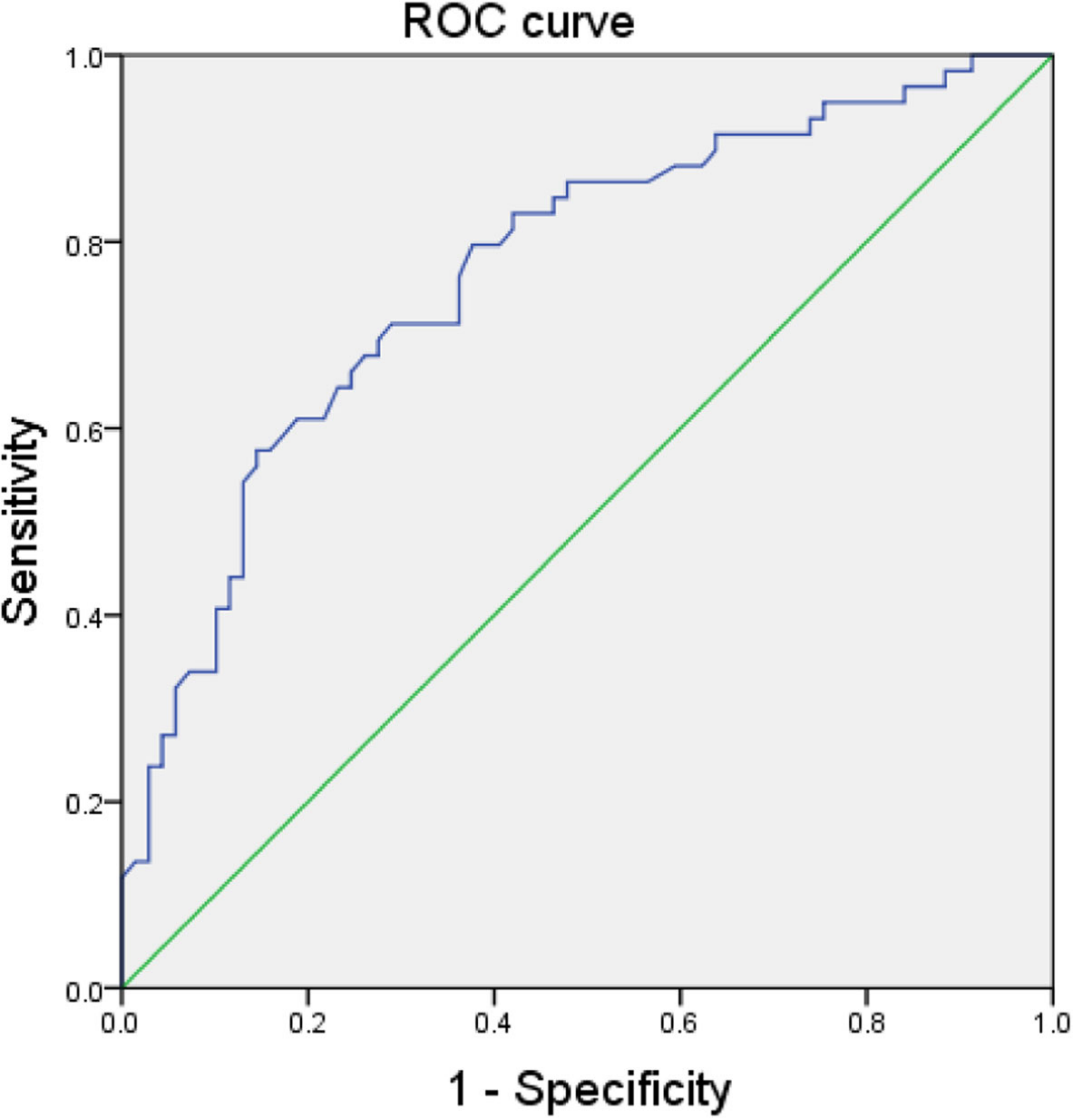
IMA predicts the ROC curve of CCC:AUC:0.769(95% CI: 0.686–0.851, *P* < 0.001), cut-off point:63.35KU/L,71.2% sensitivity,71% specificity

IMA can be used as an effective diagnostic indicator for patients with good CCC. The area under curve was 0.769 (95% CI: 0.686–0.851, *P* < 0.001),which means that the IMA level can be used to predict the good formation of CCC and have a certain accuracy. The optimal cut-off point was 63.35 KU/L, with a sensitivity of 71.2% and a specificity of 71% (Fig. 2).

## Discussion

This study shows that a high IMA level is an independent predictor of CCC and positively correlated with CCC formation. In addition, this study found a negative correlation between diabetes and CCC formation.

The formation of coronary collateral vessels is a complex process. When the blood flow of the coronary artery is severely reduced or occluded, anastomotic branches with a diameter of 20–350 μm between the coronary arteries gradually generate functional collateral circulation through a series of mechanisms under the control of end-to-side pressure of the coronary artery [2]. When coronary artery stenosis or occlusion is severe, good CCC can improve myocardial ischemia, protect myocardial contraction function, improve clinical symptoms, reduce the incidence of myocardial infarction, and reduce the size of myocardial infarction, thereby reducing the mortality associated with ischemic events and improving prognosis [3, 4, 13]. However, the determinants and influencing factors of CCC formation are also complex. The degree of CCC formation may be different in individuals with the same degree of coronary stenosis or occlusion. Studies have shown that the formation of CCC is positively correlated with the severity of coronary artery stenoses, multiple severe stenosis and duration of lesion [14]. Moreover, a large number of studies have shown that the formation of CCC is also closely related to hypertension, diabetes, metabolic syndrome, oxidative stress, endothelial dysfunction, endogenous mediators and vascular inflammation [15–17].

This study shows that a high IMA level is an independent predictor of CCC formation and is positively correlated with good CCC formation. Ischemia-modified albumin is considered a new biomarker for the assessment of myocardial ischemia that has early diagnostic value for myocardial ischemia [9]. The formation of IMA is mainly related to the oxidative stress response caused by ischemia reperfusion injury and other cardiac and extracardiac events. IMA is the product formed by the oxidation of albumin, and the higher the oxidative stress, the higher the IMA level [18, 19]. IMA is also closely related to the amount of oxygen free radicals formed during ischemia [20]. The production of IMA is also related to factors other than cardiac factors, including blood vessels, brain and skeletal muscle [7]. Previous studies have found that oxidative stress is closely related to the formation of CCC. Oxidative stress is caused by reactive oxygen species (ROS) produced in aerobic cells. The imbalance between oxidants and antioxidants in myocardial ischemia or reperfusion injury favors the production of ROS, thus resulting in oxidative stress [21]. The production of ROS is essential for the growth, proliferation and migration of vascular cells, which further promotes the formation of coronary collateral circulation [16]. Gu et al. [22] found that ROS produced by coronary ischemia or reperfusion injury play a key role in the development of CCC. Matsunaga et al. [23] demonstrated that antioxidants inhibit collateral development induced by myocardial ischemia. Demirbag et al. [24] studied oxidative stress in 176 male patients with single-vessel CTO, 94 of whom had poorly developed CCC, while 82 had well-developed CCC, the researchers found that total peroxide and the oxidative stress index were independent positive predictors of good CCC in CTO. Gök et al. [25] showed that the formation of CCC was easier when oxidative stress increased. Moreover, the increase in oxidative stress could lead to a higher level of IMA. The results of this study suggest that a high IMA level is positively correlated with CCC formation and is an independent indicator of CCC formation. The study also further confirms that in the CCC formation process, despite the involvement of various mechanisms, such as oxidative stress and oxygen radical formation, the involvement of oxidative stress may be the dominant factor in the mechanism of CCC formation.

Diabetes mellitus is an important risk factor for cardiovascular disease. This study found a negative correlation between diabetes and CCC, which may be related to the adverse effect of diabetes on coronary collateral artery formation and angiogenesis that is exerted through various mechanisms. Diabetic patients have more severe, diffuse and complex coronary atherosclerosis, which can lead to a decrease in coronary collateral artery pressure, which is not conducive to collateral formation. When diabetes mellitus develops, the parameters of vascular growth factor change, and endothelial dysfunction reduces the production of nitric oxide, which has a negative impact on the formation of neovascular intima and angiogenesis. Inflammatory cells such as neutrophils, monocytes and macrophages also play a negative role in CCC [15]. The results of this study are consistent with previous research conclusions.

At present, little research has been done on the relationship between ischemia-modified albumin and coronary collateral circulation. In this study, univariate analysis and multivariate logistic regression analysis showed that a higher IMA level is positively correlated with good CCC. The IMA level is closely related to oxidative stress during myocardial ischemia and ischemia reperfusion. Increased oxidative stress can lead to higher levels of IMA, and CCC is more likely to form when oxidative stress increases. The results of this study show that the IMA level is closely related to CCC formation and can be used as a predictor of CCC formation. However, there are still some shortcomings in this study: this study was a retrospective study, the sample size was small, no follow-up was conducted on the outcome, there are many factors influencing the formation of CCC, and a comprehensive multifactor analysis was not able to be conducted.

## Conclusions

The results of this study showed that a higher IMA level was positively correlated with good CCC, suggesting that the IMA level was closely related to good CCC and that a higher IMA level could be an effective predictor of good CCC in patients with CTO. Of course, more large-scale studies are needed to explore the relationship between ischemia-modified albumin and coronary collateral circulation.

## Data Availability

The datasets used and analyzed during the current study are available from the corresponding author on reasonable request.

## Abbreviations

IMA: Ischemia-modified albumin
CCC: Coronary collateral circulation
CTO: Chronic total occlusive disease
TBIL: Total bilirubin
LVEF: Left ventricular ejection fraction
TIMI: Thrombolysis in myocardial infarction
LAD: Left anterior descending
LCX: Left circumflex
RCA: Right coronary artery
ACCF: American College of Cardiology Foundation
AHA: American Heart Association
SCAI: The Society for Cardiovascular Angiography and Interventions
PCI: Percutaneous coronary intervention
CABG: Coronary artery bypass graft
ROS: Reactive oxygen species
